# Histatin-1 Expression in Human Lacrimal Epithelium

**DOI:** 10.1371/journal.pone.0148018

**Published:** 2016-01-29

**Authors:** Dhara Shah, Marwan Ali, Zeeshan Pasha, Assraa Jassim Jaboori, Sarmad H. Jassim, Sandeep Jain, Vinay K. Aakalu

**Affiliations:** Department of Ophthalmology and Visual Sciences, University of Illinois at Chicago, Chicago, Illinois, United States of America; Wayne State University School of Medicine, UNITED STATES

## Abstract

**Background:**

Study of human lacrimal cell biology is limited by poor access to tissue samples, heterogeneous cell composition of tissue and a lack of established lacrimal epithelial markers. In order to further our understanding of lacrimal cell biology, we sought to find a better marker for human lacrimal epithelial cells, compared to what has been reported in the literature.

**Methods:**

We utilized human Muller’s muscle conjunctival resection (MMCR) specimens containing accessory lacrimal gland (ALG) and cadaveric main lacrimal gland (MLG) as sources of lacrimal tissue. Candidate markers were sought using human ALG tissue from MMCR specimens, isolated by laser capture microdissection (LCM). Affymetrix® analysis was performed on total RNA isolated from FFPE samples to profile transcription in ALG. MMCR tissue sections were assessed by immunofluorescence using antibodies for histatin-1, lactoferrin, E-cadherin (E-cad) and alpha-smooth muscle actin (ASMA). Reverse transcriptase polymerase chain reaction (RT-PCR) analysis was performed to analyze the expression of histatin-1, E-cad and lactoferrin from cadaveric MLG.

**Results:**

Histatin-1 is expressed in ALG and MLG, localizes to lacrimal epithelium, and to a greater degree than do other putative lacrimal epithelial markers.

**Conclusions:**

Histatin-1 is a good marker for human lacrimal epithelium in ALG and MLG and can be used to identify lacrimal cells in future studies.

## Introduction

Dry eye syndrome (DES) is a multifactorial disease of the ocular surface that occurs due to tear film instability, hyperosmolarity, inflammation and loss of main and accessory lacrimal gland function. It is a common disease that affects approximately 11% to 22% of people worldwide. DES is associated with negative effects on quality of life, productivity and has a high cost of treatment [1]. Severe DES may be a debilitating condition and can lead to corneal scarring, chronic pain and blindness. Currently available therapies for DES, such as lubricating eye drops and anti-inflammatory medications, have had limited success and are primarily palliative [2]. Development of new strategies to treat DES which address loss of lacrimal function, including regenerative therapies, could provide great relief for many patients.

Understanding of lacrimal gland biology has lagged behind other fields of study and improvements in this area could drive the development of new therapeutic tools. Limitations to investigation into human lacrimal cell biology include inadequate access to tissue, heterogeneous cell populations in samples and physically small samples. In particular, the lack of well-defined cell markers for lacrimal epithelium limits the ability to isolate, identify and investigate the behavior of lacrimal epithelium *in vitro* and *in vivo* [3–5].

Previous studies have utilized a variety of proteins, such as lactoferrin (lacrimal epithelium) and alpha smooth muscle actin (ASMA) (myoepithelial cells) as presumed lacrimal markers [5–7]. However, these markers are expressed in many cell types, including those present in human tissue samples used for study of lacrimal biology [8–12]. Moreover, some studies have even utilized E-cadherin (E-cad), a known general epithelial marker, as a potential lacrimal epithelial marker [13]. Previous studies have evaluated the gene expression of accessory lacrimal gland (ALG), and have revealed a number of potential markers of interest, including histatin-1 [14].

Muller Muscle Conjunctival Resection (MMCR) surgery is a commonly used procedure in the treatment of blepharoptosis. Both MMCR surgery and the Fasanella-Servat ptosis procedure involve the resection of the posterior layers of the eyelid in the area of the ALG [15,16]. ALG tissue is noted in the surgical specimen in MMCR procedures approximately 50% of the time [15]. These surgical specimens contain conjunctiva, stromal tissue, Muller’s muscle (smooth muscle), blood vessels, nerve tissue and ALG tissue. ALG tissue contains myoepithelial cells, acinar epithelium and ductal cells.

In this study, we sought to find a reliable marker for lacrimal epithelium using Affymetrix® gene arrays, coupled with the existing knowledge from the literature on lacrimal gene expression [17]. We validated a candidate marker using primary cultures of human main lacrimal gland (MLG) cells and ALG in human MMCR surgical specimens. In addition, we evaluated candidate marker expression from cultured MLG by using reverse transcriptase polymerase chain reaction (RT-PCR).

## Materials and Methods

### Ethics Statement

Written informed consent was obtained from patients using a consent form specifically approved for this study by the Institutional Review Board (IRB) and processed by The University of Illinois at Chicago (UIC). Completed, signed consent forms were maintained according to the university guidelines following an IRB approved protocol specific for this study. Cadaver donor eye tissues, including MLG cells, included in this study were deidentified and use of these tissues was sanctioned by the UIC IRB.

### Patient Record Evaluation

Patient consent and samples were obtained using an IRB approved protocol. Inclusion criteria included age greater than or equal to 18 years old, and plans to undergo blepharoptosis repair via MMCR. Seventeen patients and twenty four eyelid specimens were included.

### Immunofluorescence Staining and Confocal Microscopy

Antibodies to histatin-1, E-cad, lactoferrin and ASMA were used for immunofluorescence staining of MMCR tissue sections. Xylene and rehydration with serial ethanol dilutions were used for deparaffinization. Slides were washed twice for 5 minutes in 0.25% Triton X-100 for permeabilization and blocked for 2 hours at room temperature with 2% BSA and 10% normal donkey serum in PBS. Slides were incubated overnight at 4°C with the primary antibody diluted in blocking solution (1:100) (Anti-Lactoferrin; Cat. #ab6410-100; Abcam, MA, USA, Anti-Histatin-1; Cat. #ab81089; Abcam, MA, USA, Anti-E-cad; Cat. #ab76055; Abcam, MA, USA and Anti-ASMA; Cat. #MS-113-P0; Thermo Scientific, MA, USA). The next day, the slides were washed twice for 5 minutes in PBS and incubated for 1–2 hours with respective secondary antibodies (Jackson ImmunoResearch laboratories INC., PA, USA) diluted in blocking solution (1:200–800). Vecta shield mounting medium with 4′,6-diamidino-2-phenylindole (DAPI; Cat. #H-1200; Vector Labs, CA, USA) was placed over the slides and covered with a glass coverslip. Slides were analyzed using the Zeiss LSM 710 Confocal Microscope.

Similarly, cultured human MLG derived epithelial cells were cultured on the chamber slides and fixed with 4% paraformaldehyde for immunostaining with antibodies to histatin-1, lactoferrin, E-cad, and ASMA.

### Laser Capture Microdissection (LCM)

LCM (Leica) was performed to isolate ALG from MMCR specimens. Briefly, 2–3 (8 μm) sections from available paraffin embedded blocks of MMCR specimens were placed on PEN membrane slides (Leica). Toluidine blue staining according to previously described protocols [18] was performed to facilitate the identification of ALGs during LCM. Multiple LCM resections of ALG from same patient slides were pooled in one tube containing Buffer PKD (RNeasy FFPE kit, QIAGEN). ALG collected in each tube was maintained within the manufacturer recommended range for subsequent RNA extraction, purification, amplification, cDNA synthesis, and transcriptional profiling.

### RNA Isolation and Purification from Micro Dissected Formalin Fixed Paraffin Embedded (FFPE) Tissue Sections

Total RNA was purified from micro dissected, FFPE samples using RNeasy from QIAGEN using the manufacturer’s protocol. Extracted RNA was analyzed using Qubit quantification and Tape Station 2200 quantification-sizing quality control system.

### Transcriptional Profiling of Laser Capture Micro Dissected FFPE Specimens

Following RNA extraction from laser capture dissected FFPE tissues, 25ng of total RNA was amplified, converted to cDNA, and labelled with “SensationPlus™ FFPE Amplification and WT Labeling Kit” from Affymetrix®. The labeled double stranded cDNA was hybridized and analyzed on “GeneChip® Human Gene 2.0 ST” Array from Affymetrix® microarray based results.

### Gene Array Data Analysis

Data obtained from Affymetrix ® microarrays were queried for expression of candidate genes of interest. Genes of interest were determined using a literature search and genes identified in glands of Wolfring [14]. In order to best utilize our limited samples, we cross referenced our gene expression results with genes found in ALG by Ubels et al in order to find overlapping expression, with regards to genes that have functional significance in lacrimal epithelium and then subcategorized these into ones that are associated with proteins secreted by lacrimal epithelium. Further queries were made to find expression levels of genes shown to be involved in lacrimal gland development [19–22] and those expressed in stem cells [23–25].

### Reverse Transcriptase Polymerase Chain Reaction (RT-PCR)

Total RNA was isolated from main human lacrimal gland tissue using RNA-easyKit (Qiagen) according to manufacturer's instruction. cDNA was synthesized from 375ng of isolated RNA using iScript cDNA synthesis kit (BioRad). The cDNA was amplified using GoTaq green master mix (Promega) using appropriate primers for histatin-1 (259bp), E-cad (291bp) and lactoferrin (251bp). GAPDH (323bp) was used as a control for intact cDNA. The PCR amplification was performed for 41 cycles at 30 sec denaturation at 95°C annealed for 30 sec at 56°C and extended for 1 min at 72°C. The amplification reaction products were resolved on 1% agarose/TAE gels, electrophoresed at 90mV and visualized by ethidium bromide staining. Primer sequences are noted in Table 1.

**Table 1:** Primer Sequences.

Primer Name	Forward Primer	Reverse Primer
GAPDH	5’-ACAGTCAGCCGCATCTTC-3’	5’-CATCGCCCACTTGATTTTG-3’
Histatin-1	5’-CGCTGATTCACATGAAAAGAGAC-3’	5’-AGGGAAGTATCATGAAACACAGA-3’
E-cadherin	5’-GAGAAACAGGATGGCTGAAGG-3’	5’-TGAGGATGGTGTAAGCGATGG-3’
Lactoferrin	5’- TGTCTTCCTCGTCCTGCTGTTCCTCG-3′	5′CTGCCTCGTATATGAAACCACCATCAA-3′

### Main Human Lacrimal Gland Epithelium Cell Culture

Human MLG primary culture was performed as follows. Freshly obtained human MLG, provided by the National Disease Research Interchange (NDRI; http://ndriresource.org/), were inspected and adventitial tissue was dissected away from the lacrimal gland tissue by an oculoplastic surgeon. The MLG was then washed with Hank’s Balanced Salt Solution (HBSS) media (Gibco) to remove any red blood cells. The tissue was cut into 2-3mm pieces (explants) using sterile forceps and scissors. MLG explants were then placed on an uncoated six well plate and 1ml HepatoSTIM ® (Corning) media supplemented with 10ng EGF 10% FBS, 2 mM L-glutamine, 2% penicillin-streptomycin. Cells extending from the explants were then grown on uncoated plates in the same media, after moving explants to other uncoated plates.

## Results

**Fig 1:**
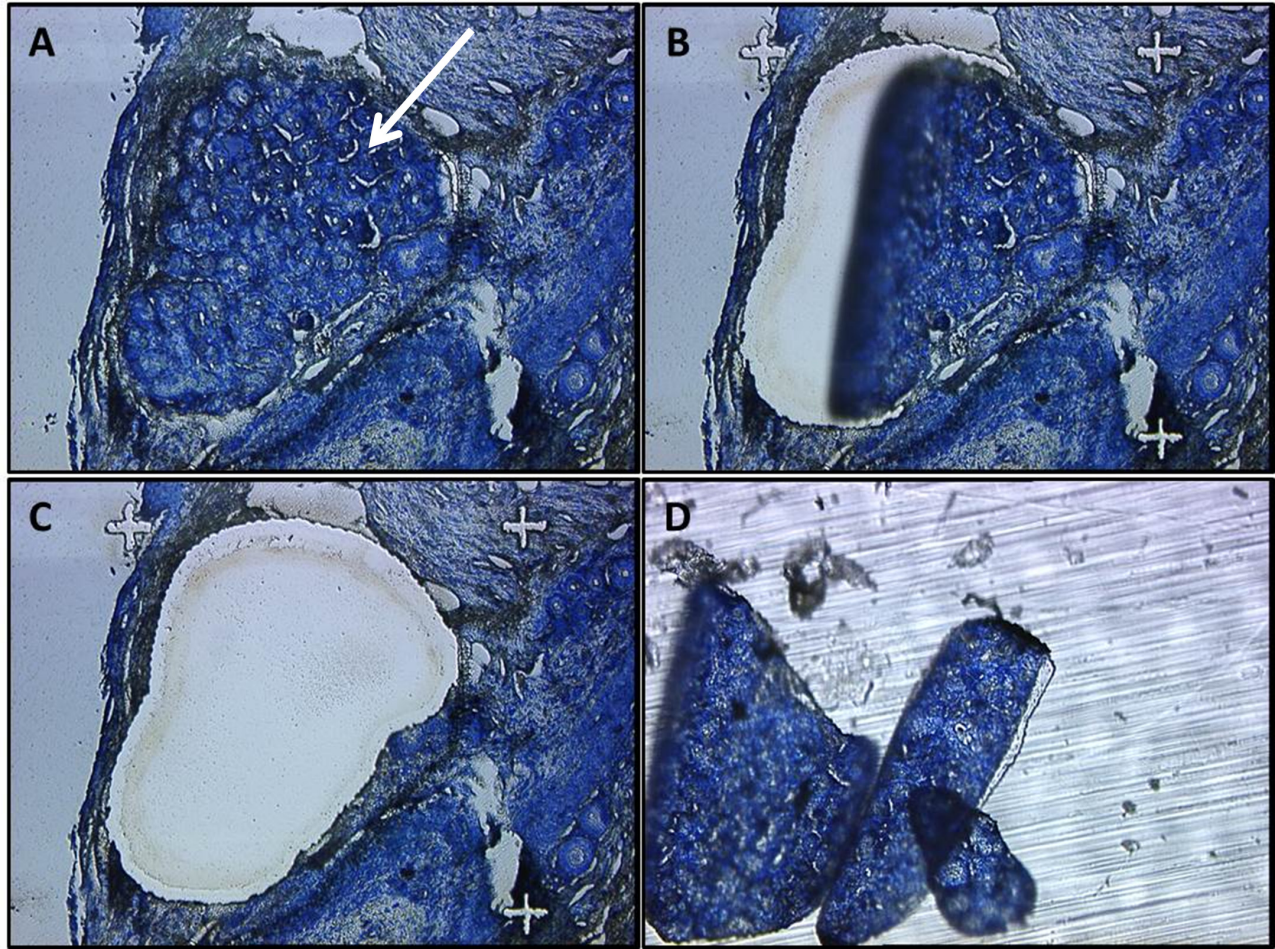
LASER Capture Microdissection of Accessory Lacrimal Gland. LCM of ALG from FFPE Human MMCR specimens. (A) Intact ALG in an MMCR specimen (white arrow). (B, C) LASER dissection of ALG from MMCR D. Isolated ALG tissue.

We determined a unique pattern of gene expression in ALG. LCM proved to be an efficient means of isolating ALG from FFPE MMCR specimens which contained multiple cell and tissue types (Fig 1). Using the “Gene Chip Human Gene 2.0 ST” Array from Affymetrix® for analysis, the genes of interest were divided into four main groups based on their expression level and functional role. Group1; includes the genes which are important to lacrimal function in ALG that include (Table 2) [14]. Group2; includes genes associated with lacrimal epithelial secreted proteins [26] that include LACRT, LYZ, and LCN1 (Table 3). Group3; includes genes shown to be important in the development of lacrimal gland [19, 21, 22], PAX6, BARX2, RUNX1-3 and FGF10 (Table 4). Group4; includes genes noted to be expressed in stem cells: NANOG, OCT4, KLF4, MYC, VIM, NES, SOX2, ABCG2, PDX1, MSI1, MSI2, PROM1, THY1, and ITGA6 (Table 5) [23–25].

**Table 2:** Important gene in ALG function.

Gene	Gene ID	Importance	Sample A	Sample B
GALNT8	26290	Lacrimal Function	5.43	5.30
PROL1	58503	Lacrimal Function	12.22	12.48
LACRT	90070	Lacrimal Function	10.93	12.58
SCGB1D1	10648	Lacrimal Function	9.98	12.20
HIST1H2AH	85235	Lacrimal Function	4.77	3.92
LYZ	4069	Lacrimal Function	11.71	12.63
LACRT	90070	Lacrimal Function	10.93	12.58
PROL1	58503	Lacrimal Function	12.22	12.48
PIGR	5284	Lacrimal Function	9.70	11.69
LCN1	3933	Lacrimal Function	12.54	13.49
LTF	4057	Lacrimal Function	10.19	11.10
PRR4	11272	Lacrimal Function	11.29	12.29
SCGB2A1	4246	Lacrimal Function	10.55	12.50
PIP	5304	Lacrimal Function	10.72	12.36

**Table 3:** Genes associated with lacrimal epithelial secreted proteins.

Gene	Gene ID	Importance	Sample A	Sample B
LACRT	90070	Secreted Lacrimal Proteins	10.93	12.58
LYZ	4069	Secreted Lacrimal Proteins	11.71	12.63
LCN1	3933	Secreted Lacrimal Proteins	12.54	13.49

**Table 4:** Genes associated with lacrimal epithelial secreted proteins.

Gene	Gene ID	Importance	Sample A	Sample B
RUNX 1	861	Lacrimal Development	7.16	7.17
RUNX 2	860	Lacrimal Development	6.05	6.142
RUNX 3	864	Lacrimal Development	6.62	6.53
PAX 6	5080	Lacrimal Development	6.98	8.12
BARX 2	8538	Lacrimal Development	7.3	8.57
FGF 10	2255	Lacrimal Development	4.81	4.72

**Table 5:** Important stem cell genes expression in ALG.

Gene	Gene ID	Importance	Sample A	Sample B
NANOG	79923	Expressed in Stem cells	6.34	5.91
OCT 4 (POU5F1)	5460	Expressed in Stem cells	6.97	6.40
KLF 4	9314	Expressed in Stem cells	5.89	5.85
MYC	4609	Expressed in Stem cells	5.72	5.89
VIM	7431	Expressed in Stem cells	7.18	8.13
NES	10763	Expressed in Stem cells	6.16	6.07
SOX 2	6657	Expressed in Stem cells	5.80	5.13
ABCG 2	9429	Expressed in Stem cells	5.10	4.53
PDX 1	3651	Expressed in Stem cells	6.09	5.65
MSI 1	4440	Expressed in Stem cells	6.87	6.05
MSI 2	124540	Expressed in Stem cells	6.31	7.06
PROM 1	8842	Expressed in Stem cells	5.47	7.41
THY 1	7070	Expressed in Stem cells	5.82	6.04
ITGA 6	3655	Expressed in Stem cells	5.64	7.03

**Fig 2:**
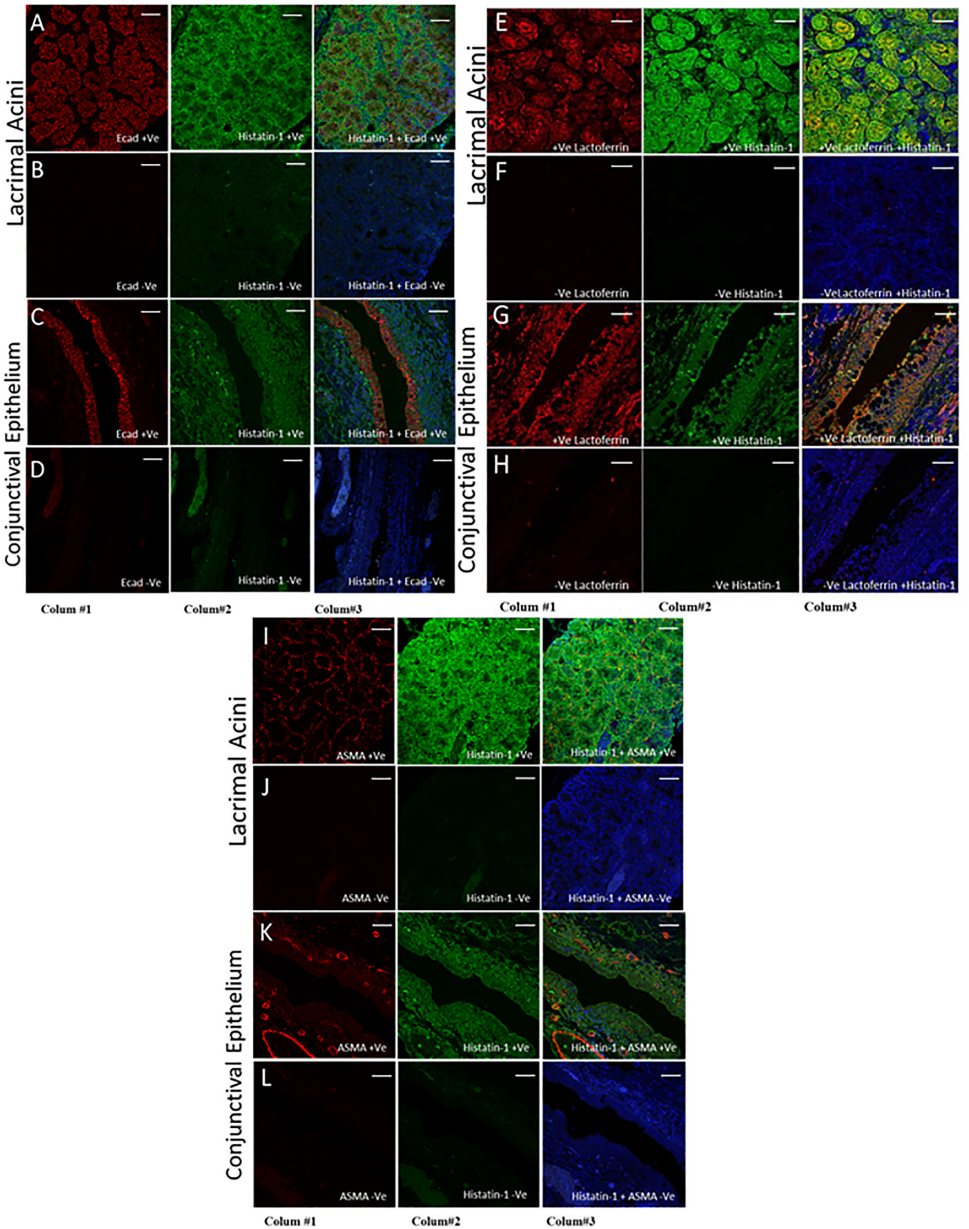
Immunofluorescence of ALG Specific Markers in MMCR. Immunofluorescence staining of ALG specific markers in MMCR specimens showing the difference in localization of markers (Ecad, Histatin-1, lactoferrin and ASMA) between lacrimal acini and conjunctival epithelium. Column 1 depicts a single stain for E-cad (Rows A-D), Lactoferrin (Rows E-H), and ASMA (Rows I-L). Column 2 shows stain for histatin-1. Column 3 shows an overlapped image of Column 1 and 2 and blue staining for nuclei (DAPI). Rows B, D, F, H, J and L are negative control images. Rows A-D show strong localization of E-cad and histatin-1 in lacrimal acini, with strong overlap and lesser localization of histatin-1 to conjunctival epithelium than lacrimal acini. In contrast, E-cad localizes to conjunctival epithelium better than it does lacrimal acini. Rows E-H show localization of lactoferrin and histatin-1 to lacrimal acini, with strong overlap and lesser localization of histatin-1 to conjunctival epithelium than to lacrimal acini. In contrast, lactoferrin localizes to conjunctival epithelium better than it does lacrimal acini. Rows I-J show good localization of ASMA to myoepithelial cells surrounding lacrimal acini, and good localization of histatin-1 to lacrimal acini without significant overlap. Rows K and L demonstrate no localization of ASMA to conjunctival epithelium by ASMA, and low levels of localization of histatin-1 to conjunctival epithelium. Scale bar, 50 μm.

**Fig 3:**
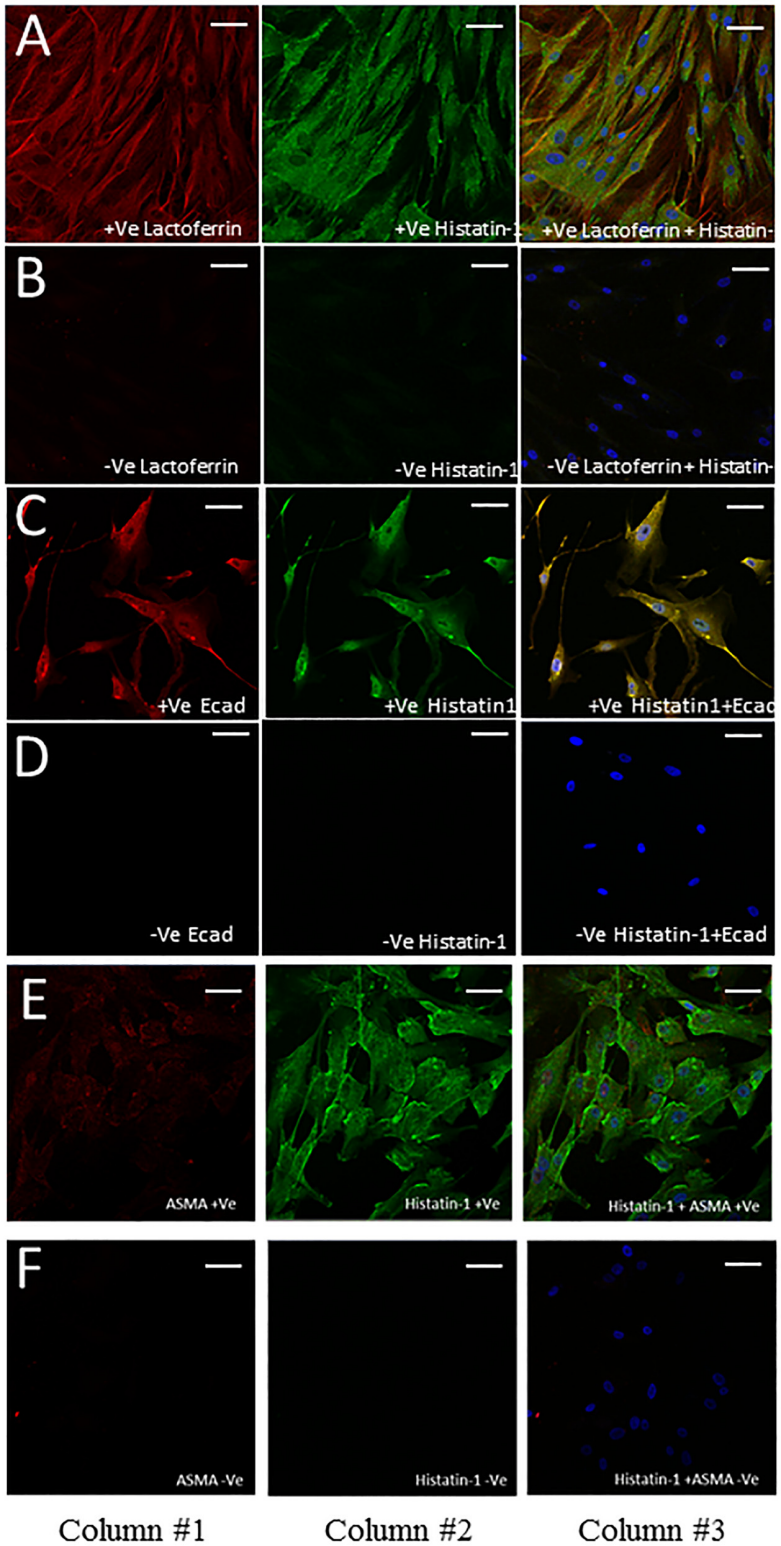
Immunofluorescence staining of cultured MLG cells. Immunofluorescence staining in cultured MLG cells using antibodies to lactoferrin (Row A), E-cad (Row C), ASMA (Row E) and histatin-1 (Rows A, C, E). Rows B, D and F are respective negative control images. Column 1 depicts a single stain for antibodies to lactoferrin (Rows A) E-cad (Rows C, D), ASMA (Rows E,F). Column 2 shows stain for histatin-1. Column 3 shows an overlapped image of Column 1 and 2 and blue staining for nuclei (DAPI). Row A shows strong localization of lactoferrin and histatin-1 to cultured MLG epithelium. Row C shows strong localization of E-cad and histatin-1 to cultured MLG epithelium. Row E shows strong localization of histain-1 to cultured MLG epithelium but ASMA does not localize well to cultured MLG epithelium. Scale bar, 50 μm.

Fig 2 shows the immunolocalization of putative markers to lacrimal acini in heterogeneous MMCR specimens. Accessory lacrimal acinar cells show strong localization of histatin-1 and relatively lower localization of lactoferrin and E-cad. ASMA localizes to the myoepithelium at the periphery of acini, without significant overlap with histatin-1. In sections showing conjunctival epithelium, E-cad and lactoferrin localize to conjunctival epithelium more than histatin-1. Fig 3 demonstrates immunostaining of lacrimal cells in culture from main lacrimal gland tissue. Histatin-1 is present in cultured lacrimal epithelium, as is E-cad and lactoferrin. Cultured lacrimal epithelium does not show localization of ASMA.

**Fig 4:**
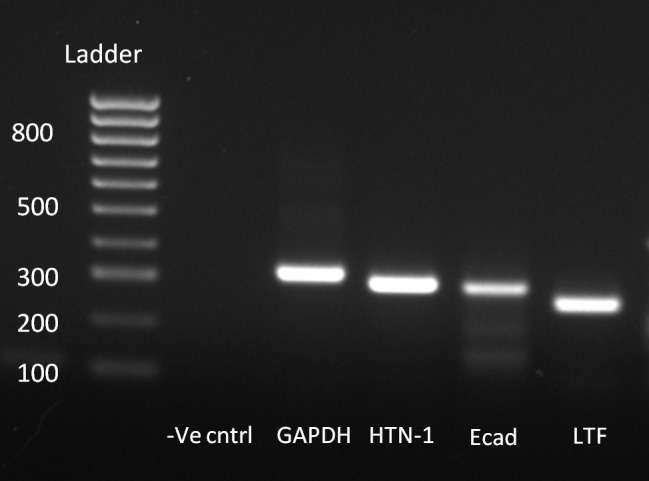
RT-PCR analysis of human main lacrimal gland tissue. RT-PCR of main lacrimal gland tissue shows expression of histatin-1, purported lacrimal markers E-cad and lactoferrin.

Finally, to confirm the expression of histatin-1 in main lacrimal gland epithelium, RT-PCR was performed. RT-PCR analysis from freshly obtained cadaveric human MLG demonstrates expression of histatin-1, lactoferrin and E-cad (Fig 4).

## Discussion

Study of human lacrimal cell biology is an important and necessary endeavor in the efforts to develop treatments for poorly understood human ophthalmic diseases, including those which may be blinding, like severe DES. These investigations are limited by the lack of defined cell markers, disease models, cell lines and available human tissue samples.

This study focused on the identification and development of a viable lacrimal epithelial cell marker to be used for future clinical studies on human lacrimal biology. We approached analysis of the transcriptome, as determined by Affymetrix®, by choosing candidate genes of interest that could be useful in future studies of lacrimal cell biology. We chose candidate genes for lacrimal epithelial markers using genes that had been identified as being important to lacrimal function in previous studies [14]. After gene expression analysis, and in the context of the existing literature, we focused on histatin-1, a member of a peptide family that has been reported in a variety of biologically interesting activities, including wound healing and microbial defense [27, 28]. We were also interested in determining the expression levels of genes shown to have import in the development of the lacrimal gland [19–22]. Greater knowledge of the expression of these genes may be useful in future developmental biology studies or in directed differentiation experiments. Finally, given the need for new therapeutics and the exciting potential of regenerative medicine efforts, we sought to determine the expression of genes known to be important in stem cell biology [23–25].

Previous studies note the expression of histatin-1 in ALG [18] and, minimally on the ocular surface, though there are conflicting results regarding expression on the ocular surface [29–32]. Our study corroborates the high expression of histatin-1 in isolated human ALG tissue. We extended this analysis to validate the utility of histatin-1 as a lacrimal epithelial cell marker by demonstrating the expression of histatin-1 in cultured lacrimal cells, for the first time. Moreover, we found that histatin-1 localizes to lacrimal acinar cells more strongly than surrounding conjunctival epithelial cells in MMCR specimens. Taken together, these data support the hypothesis that histatin-1 is a useful marker of lacrimal epithelium.

The histatin family of peptides consists of two genes (HTN1, HTN3) and numerous peptides translated from these genes [33]. HTN1 produces the proteins histatin-1 and 2, with histatin-2 being an unphosphorylated variant of histatin-1[33]. Histatin genes are expressed only in primates [34]. Histatins are well described in the saliva and have numerous biological activities including anti-fungal properties, wound healing promotion, among others [33]. In addition, these histatin peptides have been found to be expressed, at low levels, on the ocular surface [29–31]. Until now, to our knowledge, histatin proteins have not been found in the tear proteome [35].

Although histatin-1 localizes well to lacrimal epithelium and is expressed at high levels in both ALG and MLG, it is also present in conjunctival epithelium. All the putative lacrimal epithelial markers used in this study show strong overlap of localization in both conjunctival and lacrimal epithelial cells. We believe histatin-1 has the least amount of co-localization to conjunctival epithelium, as compared with lactoferrin and E-cad. ASMA seems to only identify myoepithelial cells in these samples. Thus, although histatin-1 may be a useful marker for future studies in lacrimal cell biology, there are limits to its ability to differentiate among all cell types. The development of markers to identify sub populations of cells in heterogeneous samples is often limited by the lack of absolutely specific markers and thus must be used in a relative manner based on context. We suggest the use of multiple cell markers and different types of assays in future studies on lacrimal cell biology in order to validate and characterize cell types.

It is interesting to note that our Affymetrix® gene microarray analysis of ALG shows the high expression of a number of developmental and stem cell genes which are significant and relevant to future studies. These cells express a number of the baseline markers of pluripotency including Oct4, Sox2, Klf4, c-Myc. This may indicate the suitability of these cells for use in development of regenerative therapies for DES.

Limitations of this study include small specimens, and few available samples. Furthermore, previously reported studies demonstrated only low levels of expression of histatin-1 on the ocular surface, and tear proteomic experiments did not demonstrate histatin family members in the tear film. These results may indicate that histatin proteins are not secreted onto the ocular surface, or may reveal the limitations and variability of body fluid proteomic analysis [35]. It may also be the case that histatins are secreted in response to environmental factors or at different times of day, as has been shown in saliva [36–38]. These issues underline the importance of future larger scale studies.

Future studies on lacrimal epithelium to investigate human disease processes, including DES, may utilize histatin-1 as a marker to identify changes in lacrimal cell function or biology. Moreover, the presence of stem cell markers noted in our gene expression array analysis are encouraging and should be investigated in future experiments. Finally, future studies investigating the structure and function of histatin-1 in ocular tissues will need to be considered. Thus, we have demonstrated that histatin-1 is a useful marker of lacrimal epithelium for future studies of lacrimal cell biology and function.

## References

[1] Brewitt H, Sistani F. Dry eye disease: the scale of the problem. Surv Ophthalmol. 2001;45 Suppl 2:S199–202. Review 11587143 10.1016/s0039-6257(00)00202-2

[2] Tiwari S, Ali MJ, Vemuganti GK. Human lacrimal gland regeneration: Perspectives and review of literature. Saudi J Ophthalmol. 2014;1:12–8. 10.1016/j.sjopt.2013.09.004 PMC3923198 24526853

[3] Liu A, Cheng L, Du J, Peng Y, Allan RW, Wei L et al Diagnostic utility of novel stem cell markers SALL4, OCT4, NANOG, SOX2, UTF1, and TCL1 in primary mediastinal germ cell tumors. Am J Surg Pathol. 2010;34(5):697–706. 10.1097/PAS.0b013e3181db84aa 20410807

[4] Tiwari S, Ali MJ, Balla MM, Naik MN, Honavar SG, Reddy VA, et al Establishing human lacrimal gland cultures with secretory function. PLoS One. 2012;7(1):e29458 10.1371/journal.pone.0029458 Epub 2012 Jan 13. 22253725 PMC3258235

[5] Spaniol K, Metzger M, Roth M, Greve B, Mertsch S, Geerling G, et al Engineering of a secretory active three-dimensional lacrimal gland construct on the basis of decellularized lacrimal gland tissue. Tissue Eng Part A. 2015;21(19–20):2605–17. 10.1089/ten.TEA.2014.0694 26222647

[6] Shatos MA, Haugaard-Kedstrom L, Hodges RR, Dartt DA. Isolation and characterization of progenitor cells in uninjured, adult rat lacrimal gland. Invest Ophthalmol Vis Sci. 2012;6:2749–59. 10.1167/iovs.11-9025 PMC3367467 22427571

[7] Wilk CM, Vigneswaran N, Heese A, Hornstein OP, Naumann GO. Immunohistochemical characterization of epithelial cells in human lacrimal glands. II. Inflammatory and neoplastic lesions of lacrimal glands. Graefes Arch Clin Exp Ophthalmol. 1990;1:65–72. 10.1007/BF02764294 1690161

[8] Stoeckelhuber M1, Scherer EQ, Janssen KP, Slotta-Huspenina J, Loeffelbein DJ, Rohleder NH, et al The human submandibular gland: immunohistochemical analysis of SNAREs and cytoskeletal proteins. J Histochem Cytochem. 2012;60(2):110–20. 10.1369/0022155411432785 22131313 PMC3351120

[9] Si Y, Wang J, Guan J, Han Q, Hui Y. Platelet-derived growth factor induced alpha-smooth muscle actin expression by human retinal pigment epithelium cell. J Ocul Pharmacol Ther. 2013;3:310–8. 10.1089/jop.2012.0137 23116162

[10] De Saint Jean M, Baudouin C, Di Nolfo M, Roman S, Lozato P, Warnet JM, et al Comparison of morphological and functional characteristics of primary-cultured human conjunctival epithelium and of Wong-Kilbourne derivative of Chang conjunctival cell line. Exp Eye Res. 2004;2:257–74. 10.1016/j.exer.2003.10.006 14729358

[11] Santagati MG1, La Terra Mulè S, Amico C, Pistone M, Rusciano D, Enea V. Lactoferrin expression by bovine ocular surface epithelia: a primary cell culture model to study lactoferrin gene promoter activity. Ophthalmic Res. 2005;5:270–8. Epub 2005 Aug 9. 10.1159/000087372 16103737

[12] Flanagan JL, Willcox MD. Role of lactoferrin in the tear film. Biochimie. 2009;1:35–43. 10.1016/j.biochi.2008.07.007 Epub 2008 Jul 31. Review. 18718499

[13] Hirayama M1, Ogawa M, Oshima M, Sekine Y, Ishida K, Yamashita K, et al Functional lacrimal gland regeneration by transplantation of a bioengineered organ germ. Nat Commun. 2013;4:2497 10.1038/ncomms3497 24084941 PMC3806342

[14] Ubels JL, Gipson IK, Spurr-Michaud SJ, Tisdale AS, Van Dyken RE, Hatton MP. Gene expression in human accessory lacrimal glands of Wolfring. Invest Ophthalmol Vis Sci. 2012;53(11):6738–47. 10.1167/iovs.12-10750 22956620 PMC4113189

[15] Kulchaiyawat V, Aakalu V K, Sajja K, Gupta S, Hallak J, Setabutr P. A clinicopathological correlation between müller’s muscleconjunctival resection and corneal staining pattern (abstract). ARVO 2010 Available: http://iovs.arvojournals.org/article.aspx?articleid=2370165&resultClick=1.

[16] Buckman G, Jakobiec FA, Hyde K, Lisman RD, Hornblass A, Harrison W, et al Success of the Fasanella-Servat operation independent of muller’s smooth muscle excision.Ophthalmology. 1989;96(4): 413–8. 2726170 10.1016/s0161-6420(89)32876-4

[17] Hodges RR, Dartt DA. Regulatory pathways in lacrimal gland epithelium. Int Rev Cytol. 2003;231:129–96. 14713005 10.1016/s0074-7696(03)31004-6

[18] Ubels JL, Gipson IK, Spurr-Michaud SJ, Tisdale AS, Van Dyken RE, Hatton MP. Gene expression in human accessory lacrimal glands of Wolfring. Invest Ophthalmol Vis Sci. 2012;11:6738–47. 10.1167/iovs.12-10750 PMC4113189 22956620

[19] Makarenkova HP, Ito M, Govindarajan V, Faber SC, Sun L, McMahon G, et al FGF10 is an inducer and Pax6 a competence factor for lacrimal gland development. Development. 2000;127(12):2563–72. 10821755 10.1242/dev.127.12.2563

[20] Grishina IB, Kim SY, Ferrara C, Makarenkova HP, Walden PD. BMP7 inhibits branching morphogenesis in the prostate gland and interferes with Notch signaling. Dev Biol. 2005;288(2):334–47. 16324690 10.1016/j.ydbio.2005.08.018 PMC2644052

[21] Tsau C, Ito M, Gromova A, Hoffman MP, Meech R, Makarenkova HP. Barx2 and Fgf10 regulate ocular glands branching morphogenesis by controlling extracellular matrix remodeling. Development. 2011;138(15):3307–17. 10.1242/dev.066241 21750040 PMC3133920

[22] Voronov D, Gromova A, Liu D, Zoukhri D, Medvinsky A, Meech R, et al Transcription factors Runx1 to 3 are expressed in the lacrimal gland epithelium and are involved in regulation of gland morphogenesis and regeneration.Invest Ophthalmol Vis Sci. 2013;54(5):3115–25. 10.1167/iovs.13-11791 23532528 PMC3643397

[23] Liu X1, Huang J, Chen T, Wang Y, Xin S, Li J, et al Yamanaka factors critically regulate the developmental signaling network in mouse embryonic stem cells. Cell Res. 2008;12:1177–89. 10.1038/cr.2008.309 19030024

[24] Takahashi K, Tanabe K, Ohnuki M, Narita M, Ichisaka T, Tomoda K, et al Induction of pluripotent stem cells from adult human fibroblasts by defined factors. Cell. 2007;131(5):861–72. 18035408 10.1016/j.cell.2007.11.019

[25] Bhattacharya B, Miura T, Brandenberger R, Mejido J, Luo Y, Miura T, et al Gene expression in human embryonic stem cell lines: unique molecular signature. Blood. 2004;103(8):2956–64. 15070671 10.1182/blood-2003-09-3314

[26] Tiwari S, Ali MJ, Vemuganti GK. Human lacrimal gland regeneration: Perspectives and review of literature. Saudi J Ophthalmol. 2014;28(1):12–8. 10.1016/j.sjopt.2013.09.004 24526853 PMC3923198

[27] Oudhoff MJ, Kroeze KL, Nazmi K, van den Keijbus PA, van 't Hof W, Fernandez-Borja M, et al Structure-activity analysis of histatin, a potent wound healing peptide from human saliva: cyclization of histatin potentiates molar activity 1,000-fold. FASEB J. 2009;11:3928–35. 10.1096/fj.09-137588 19652025

[28] Oudhoff MJ, Blaauboer ME, Nazmi K, Scheres N, Bolscher JG, Veerman EC. The role of salivary histatin and the human cathelicidin LL-37 in wound healing and innate immunity. Biol Chem. 2010;5:541–8. 10.1515/BC.2010.057 20302519

[29] Jumblatt MM, Imbert Y, Young WW Jr, Foulks GN, Steele PS, Demuth DR. Glycoprotein 340 in normal human ocular surface tissues and tear film. Infect Immun. 2006;74(7): 4058–63. 16790779 10.1128/IAI.01951-05 PMC1489741

[30] Steele PS, Jumblatt MM. Defense proteins of the ocular surface (abstract). ARVO 2004 Available: http://iovs.arvojournals.org/article.aspx?articleid=2409326.

[31] Steele PS, Jumblatt MM, Smith NB, Pierce WM. Detection of histatin 5 in normal human Schirmer strip samples by mass spectroscopy (abstract). ARVO 2002, Available: http://iovs.arvojournals.org/article.aspx?articleid=2417916.

[32] Huang LC, Jean D, Proske RJ, Reins RY, McDermott AM. Ocular surface expression and in vitro activity of antimicrobial peptides. Curr Eye Res. 2007;7–8:595–609. 10.1080/02713680701446653 PMC2430515 17852183

[33] Melino S, Santone C, Di Nardo P, Sarkar B. Histatins: salivary peptides with copper (II)- and zinc(II)-binding motifs: perspectives for biomedical applications. FEBS J. 2014;3:657–72. 10.1111/febs.12612 24219363

[34] Kavanagh K, Dowd S. Histatins: antimicrobial peptides with therapeutic potential.J Pharm Pharmacol. 2004;56(3):285–9. 15025852 10.1211/0022357022971

[35] Perumal N, Funke S, Wolters D, Pfeiffer N, Grus FH. Characterization of human reflex tear proteome reveals high expression of lacrimal proline-rich protein 4 (PRR4). Proteomics. 2015;15(19):3370–81. 10.1002/pmic.201400239 26173177

[36] Gusman H1, Leone C, Helmerhorst EJ, Nunn M, Flora B, Trpxler RF, et al Human salivary gland-specific daily variations in histatin concentrations determined by a novel quantitation technique. Arch Oral Biol. 2004; 1:11–22. 10.1016/s0003-9969(03)00182-1 14693192

[37] Campese M, Sun X, Bosch JA, Oppenheim FG, Helmerhorst EJ. Concentration and fate of histatins and acidic proline-rich proteins in the oral environment. Arch Oral Biol. 2009;4:345–53. 10.1016/j.archoralbio.2008.11.010 PMC2680473 19159863

[38] Manconi B, Cabras T, Pisano E, Sanna MT, Olianas A, Fanos V, et al Modifications of the acidic soluble salivary proteome in human children from birth to the age of 48months investigated by a top-down HPLC-ESI-MS platform. J Proteomics. 2013;91:536–43. 10.1016/j.jprot.2013.08.009 23973467

